# Distinguish water utilization strategies of trees growing on earth‐rocky mountainous area with transpiration and water isotopes

**DOI:** 10.1002/ece3.3584

**Published:** 2017-11-05

**Authors:** Guodong Jia, Ziqiang Liu, Lixin Chen, Xinxiao Yu

**Affiliations:** ^1^ Key Laboratory of State Forestry Administration on Soil and Water Conservation Beijing Forestry University Beijing China; ^2^ Beijing Engineering Research Center of Soil and Water Conservation Beijing Forestry University Beijing China; ^3^ School of Soil and Water Conservation Beijing Forestry University Beijing China

**Keywords:** IsoSource mixing model, species comparison, stable isotope, transpiration, water use patterns

## Abstract

Water stress is regarded as a global challenge to forests. Unlike other water‐limited areas, the water use strategies of rocky mountainous forests, which play an important ecohydrological role, have not received sufficient attention. To prove our hypothesis that species adopt different water use strategies to avoid competition of limited water resources, we used site abiotic monitoring, sap flow and stable isotope method to study the biophysiological responses and water use preferences of two commonly distributed forest species, *Pinus tabuliformis* (Pt) and *Quercus variabilis* (Qv). The results showed that Pt transpired higher than Qv. Pt was also prone to adopt isohydric water use strategy as it demonstrated sensitive stomatal control over water loss through transpiration. Qv developed cavitation which was reflected by the dropping *E*_c_ in response to high vapor pressure deficit, concentrated peak sap flux density (*J*_s_), and enlarged hysteresis loop. Considering the average soil depth of 52.8 cm on the site, a common strategy shared by both species was the ability to tap water from deep soil layers (below 40 cm) when soil water was limited, and this contributed to the whole growing season transpiration. The contribution of surface layer water to plant water use increased and became the main water source for transpiration after rainfall. Qv was more efficient at using water from surface layer than Pt due to the developed surface root system when soil water content was not stressed. Our study proves that different water‐using strategies of co‐occurring species may be conducive to avoid competition of limited water resources to guarantee their survival. Knowledge of water stress‐coping strategies of trees has implications for the understanding and prediction of vegetation composition in similar areas and can facilitate forest management criteria for plantations.

## 1 INTRODUCTION

Forest ecosystem plays an important role in maintaining water and controlling soil erosion worldwide. The survival of forest ecosystem is severely challenged under scenarios such as global surface air temperature increase and precipitation reduction in northern China (Shi et al., 2014), which would greatly affect vegetation composition and plant water use. Currently, climate‐induced forest degradation has already become a global occurrence (Allen et al., 2010). Given the important role of water yield and anti‐erosion of forests, especially in drought‐prone mountainous areas, study of their survival strategies under such circumstances constitutes a vital issue for guaranteeing a safe ecological environment for human society in the future.

Pines and oaks are widely distributed globally and tend to co‐occur in water‐limited systems (Nowacki & Abrams, 2008), but they are different in anatomical and physiological features, such as leaf types and hydraulic systems, that lead to different hydraulic regulations of transpiration to atmospheric and soil drought, such as vapor pressure deficit (VPD) (Domec et al., 2015) and soil dryness (O'Grady, Worledge, & Battaglia, 2008). Pines have small, evergreen needle leaves and rely on smaller diameter tracheids for water transport. On the other hand, oaks are mostly deciduous and rely on large‐diameter vessels for water transport (Renninger, Carlo, Clark, & Schäfer, 2015). Consequently, ring‐porous species like oaks have been found to have fewer vessels per unit sapwood but higher leaf‐specific hydraulic conductivity than conifers, like pines (McCulloh et al., 2010), but the different size of water‐transport conduit of the two species may indicate different abilities to regulate water loss and to minimize fluctuations in tissue water potential, that is, the larger the conduits are the more vulnerable, the species is to embolism. The existing observations are mixed. For example, in terms of hydraulic regulation, several studies have found that like other ring‐porous species, oaks tend to be more anisohydric, allowing the leaf water potentials to drop with increasing evaporative demand and drought stress (Martínez‐Vilalta, Poyatos, Aguadé, Retana, & Mencuccini, 2014). By contrast, a global synthesis found that conifers had the largest “safety margin” between minimum in situ water potential and the water potential at which 50% of hydraulic conductivity is lost to embolism (Choat et al., 2012). However, in another synthesis study, oaks and pines are both proved to be partial isohydric (Martínez‐Vilalta et al., 2014), showing strong stomatal control of transpiration rate, and thus maintain a stable leaf water potential regardless of soil water availability. Also, species differ in terms of resource acquisition strategies for water and nutrients, particularly in ecosystems with soils that exhibit low water and nutrient‐holding capacity in the upper soil layers (Forrester, Theiveyanathan, Collopy, & Marcar, 2010). In this respect, stable isotopes provide a powerful tool in identifying plant water sources. There is no isotope fractionation during water uptake by terrestrial plants (Dawson, Mambelli, Plamboeck, Templer, & Tu, 2002). Therefore, comparisons of stable isotopic compositions of plant stem water with those of the potential water sources (e.g., soil water from varying depths and groundwater) could identify the most probable sources of water transpired by plants.

More diverse communities have potential to better resist future drought (Jactel et al., 2009). Although the protective forests were historically widespread in North China, yet the knowledge gaps on plant functioning and in turn on plant resilience to extreme drought have not been filled. Therefore, in this study, we focused on investigating the integrated biophysiological strategies of these two important coexisting tree species, namely *Pinus tabuliformis*, a conifer that is widespread across the world, and *Quercus variabilis*, a common native oak species in mountainous regions in China, with the combination of sap flow measurements and isotopic examination. We hypothesized that the water use of codominant species would stratify to minimize the negative impact from competition over limited water resource. Specifically, we (1) investigated the species‐specific responses of canopy transpiration to VPD and soil water availability under different rainfall regimes and (2) determined their water sources during rainless intervals using stable isotope methods. It is critical to understand the water use characteristics and physiological responses and it will provide new and powerful tools for selection of species in future forest management in this area.

## 2 MATERIALS AND METHODS

### 2.1 Site description

Jiufeng National Forest Park (40°3.511′N, 116°5.242′E) is located in the western mountain range in the northwestern region of Beijing. It covers an area of 832.04 hectares with a forest coverage rate of 96.4%. The park is 30 km away from urban Beijing. Observation over the recent 10 years showed that the annual average temperature is 12.5°C, with the highest record of 41.6°C (July) and the lowest record of −19.6°C (January). The average annual precipitation during 2006–2016 was 630 mm with 74% occurred in summer from June to September. The annual potential evaporation ranges from 1,800 to 2,000 mm. Frost usually occurs from the beginning of September and ends in the beginning of April. There are several locations where natural springs occur. The underlying parental material is limestone, and the average depth of the soil is 52.8 cm, ranging from 20 to 114 cm, with 61% distributed in the range of 30–59 cm. The gravel content is high (up to 85% of gravel smaller than 2 mm). Given the thin soil layer, we classified the surface layer as the top 30 cm and the deep layer as depths below 40 cm. The overstory of the whole mountain area is predominantly composed of *P. tabuliformis* (19.1%), *Q. variabilis* (26.2%), *Platycladus orientalis* (17.3%), and *Robinia pseudoacacia* (3.9%). We selected four plots of similar topography (20 × 20 m each) codominated by *P. tabuliformis* and *Q. variabilis*, which also served as a representation of coniferous and broadleaf species, to study the underlying dynamics that enable forest survival under drought conditions. Detailed plot characteristics are presented in Table 1. To get the information of root biomass distribution along the soil profile, ten trees were selected. Three pits were dug to the depth of approximately 120 cm (weathered bedrock) around each sampled tree. Soil cubes were excavated at every 20‐cm interval. No roots were found below 60 cm. The pit was refilled after sampling. The roots were sieved out of the soil, and their diameters were measured with a vernier caliper. The roots then were classified into three diameter classes: >5, 2–5, and <2 mm. The sorted roots were sealed in plastic bags and transported to the laboratory to determine the dry biomass (roots were dried in an oven at a temperature of 105°C for 48 hr).

**Table 1:** Survey of the plots

Plot no.	Species	Topography	Total number of stems	Mean tree height (m)	Mean DBH (cm)	Transpiration sample number	Mean DBH of transpiration samples (cm)
1	*Pinus tabuliformis*	Altitude (m): 135slope direction: semi‐sunny slopesoil depth (cm): 53(4.6)	45	12.45 (3.2)	18.6 (3)	14	18.1 (4.2)
	*Quercus variabilis*		58	15.69 (5.4)	23.5 (4.4)	14	23.1 (5.3)
2	*Pinus tabuliformis*	Altitude (m): 139slope direction: semi‐sunny slopesoil depth (cm): 51(3.5)	54	10.45 (2.2)	16.6 (2)	8	16.2 (3.5)
	*Quercus variabilis*		49	14.28 (5.1)	21.5 (3.2)	8	21.2 (4.5)
3	*Pinus tabuliformis*	Altitude (m): 129slope direction: semi‐sunny slopesoil depth (cm): 49(2.8)	40	9.45 (1.2)	15.6 (2)	7	15.1 (2.1)
	*Quercus variabilis*		48	13.69 (2.4)	20.3 (3.4)	7	20.4 (3.1)
4	*Pinus tabuliformis*	Altitude (m): 126slope direction: semi‐sunny slopesoil depth (cm): 50(4.1)	46	11.58 (3.2)	16.7 (1.9)	12	16.3 (3.6)
	*Quercus variabilis*		53	15.69 (5.4)	20.5 (2.4)	13	20.1 (2.1)

The data are presented as the mean (*SD*).

### 2.2 Site abiotic monitoring

We used a HOBO U30 meteorological station (Onset Computer Corp., Boune, MA, USA) to monitor the solar radiation (W/m^2^), temperature (°C), relative humidity (%), wind (m/s), precipitation (mm), and potential evapotranspiration (PET) adjacent to the plot from 1 January 2014 to 31 December 2015. The data were measured every 10 seconds and recorded automatically as 30‐min averages.

EM50 probes (Decagon Devices Inc., Pullman, WA, USA) were deployed at the same locations as the HOBO U30 to continuously monitor soil water (cm^3^/cm^3^). The probes were only inserted into the soil at depths of −20 and −40 cm. The deeper layers were not monitored consecutively during the whole experiment period because the rocky substrate of the deeper layers failed to guarantee the close contact between the probes and the soil, and this would yield inaccurate observation data, but they were examined with isotopic samplings when the soil excavation was deep to 60 cm. To compare across plots and to eliminate the influence of soil texture heterogeneity on soil water content, drought intensity is best quantified in the form of relative extractable soil water (REW, dimensionless) over the whole monitoring zone (Gartner, Nadezhdina, Englisch, Cermak, & Leitgeb, 2009; Tognetti et al., 2009).

(1) $$\text{REW}=\left(\theta -\theta_{\min}\right)/\left(\theta_{\max}-\theta_{\min}\right)$$

where θ_max_ and θ_min_ are the respective averaged maximum and minimum measured soil water contents (θ) across the two monitored layers (0–20 cm and 20–40 cm) during the observation period. REW varies between 0 and 1. REW below 0.4 has been widely considered as a soil water stress condition (Granier, Bréda, Biron, & Villette, 1999) (Bréda, Huc, Granier, & Dreyer, 2006).

### 2.3 Canopy transpiration and conductance

Sap flow provides insights into environmental limitations, but it has been commonly transformed to canopy transpiration because (1) canopy transpiration can be compared directly with rainfall and thus facilitates the understanding of the stand water balance status, (2) the water use of the whole forest ecosystem can be estimated and compared with other studies (Wilson, Hanson, Mulholland, Baldocchi, & Wullschleger, 2001), and (3) canopy conductance which is estimated from canopy transpiration serves as importance factor in describing trees water control. Sap flux observations were conducted from DOY 94 to DOY 305 in 2014 and 2015. Trees were classified into different DBH classes by every 2‐cm increments. The measurements were made on a minimum of seven trees of each species that were randomly selected from each DBH class (Table 1). Each DBH class was represented with at least one sample tree. Thermal dissipation probes (10 and 20 mm long; Dynamax Inc.*,* Houston, TX, USA) were installed under the bark and cambium at DBH level (1.3 m above the soil). Data were collected every ten seconds using a CR1000 data logger (Campbell Scientific Inc., Logan, UT, USA), and 30‐min averages of temperature difference data were stored. The sensor signal was converted to sap flux density (*J*_s_, g m^−2^ s^−1^) according to Granier (Granier, 1987). Drift of daily value of maximum temperature differences (*DT*_max_) between the two probes was reduced according to the proved procedures (Lu, Urban, & Zhao, 2004). Both species had thin sapwood depth, and close sample points (10 and 30 mm) would not improve the estimate (Ford, McGuire, Mitchell, & Teskey, 2004). Therefore, we did not conduct the measurement of sap flow radial profile due to limited probes.

The upscaling included two parts: spatial upscaling from individual sample tree to the stand transpiration and temporal upscaling from second to daily value (Matheny et al., 2014). Spatially, because *J*_s_ of sampled trees was used as a representation of the corresponding DBH class, the sap flux of certain DBH class was calculated by multiplying the average *J*_s_ of sampled trees to As of trees within this DBH class and then summing the value of these individual trees. To get stand sap flux, we summed the values of individual DBH classes. As to the temporal upscaling, to get daily sap flux (kg/day), we first calculated the half‐hourly whole tree sap flux by multiplying the product of *J*_s_ and As with a time conversion coefficient to convert from seconds to a half‐hour value (1,800), and then summed these values for a 24‐hr period. Daily sap flux was converted to stand‐scale canopy transpiration (*E*_c_, in mm/day) by dividing the plot area.

Sapwood depth was determined by visual inspection of the increment core to determine the average sapwood depth across the plots. We chose probes shorter than the sapwood depth to avoid insertion into heartwood which would result in underestimation of transpiration (Hultine et al., 2010), and the sapwood area (As) was calculated as total basal area minus the heartwood area. The sapwood area of the trees without core sampling was estimated from the relationship between As and DBH (diameter at breast height), established from the cored trees.

(2) $${\text{As}}_{\mathrm{Pinus}}=0.5{\text{DBH}}^{1.9}\;\left(R^{2}=0.98,\;n=26\right)$$

(3) $${\text{As}}_{\mathrm{Quecus}}=15\ln\text{DBH}-14.2\;\left(R^{2}=0.86,\;n=26\right)$$

The canopy conductance was derived following the Penman–Monteith equation (Zeppel et al., 2008)

(4) $$G_{c}=\frac{G_{a}\lambda E\gamma }{\Delta R_{n}+3,600\rho C_{p}\text{VPD}G_{a}-\lambda E\left(\Delta +\gamma \right)}$$

where *k* is the latent heat of vaporization of water (2.39 MJ/kg), *E*_c_ is transpiration by species expressed as mm/hr, ∆ is the ratio of the saturated vapor pressure to temperature (kPa/C), *R*_n_ is the net radiation (MJ m^−2^ hr^−1^), 3,600 is the conversion factor from seconds to hours, *q* is air density (kg/m^3^), *C*_p_ is the specific heat of air (1.013 MJ kg^−1^ C^−1^), VPD is the vapor pressure deficit (kPa), c is the psychrometric constant (0.066 kPa/C), and *G*_a_ is the aerodynamic conductance (m/s) calculated from Equation (5) (Mielke et al., 1999).

(5) $$G_{a}=\frac{k^{2}*u}{{\left[\ln\left(\frac{z-d}{z_{0}}\right)\right]}^{2}}$$

where *k* is von Karman's constant, 0.41, *u* is the wind speed above the canopy (m/s), *Z* is the reference height at which wind is recorded (m), *Z*_0_ is the roughness height (usually 0.1 hr, and *h* is the canopy height), and *d* is the displacement height (0.7 hr). The wind speed above the canopy was measured as 30‐min averages; therefore, stability effects were included and corrections are not required.

### 2.4 Isotopic sampling and measurement

Sample collection and treatment. (1) Plant samples: Plant tissue samples were collected once a week in each month (four times a month), from April to October, both in 2014 and 2015. Fully matured and suberized twigs (diameter 0.3–0.5 cm, length 3–5 cm) at 3–4 m aboveground were sampled from each of the four cardinal directions of the trees when possible. Then, they were placed into capped vials immediately upon collection and sealed with Parafilm “M” (Bemis NA Co., Wisconsin, USA). Samples were maintained in a cooler with ice in the field and kept frozen (−20 °C) in the laboratory prior to water extraction. (2) Soil samples: Soil samples were collected together with plant sampling. Soil samples were collected with a soil auger at the depths of 0–10, 10–20, 20–30, 30–40, and 40–60 cm. Besides, a round of soil water sampling down to the depth of 60 cm was conducted to enhance the vertical and horizontal representativeness as the supplement to the consecutive EM50 monitoring of soil water (described in Section 2.2). For each depth, three replicate soil samples were collected around the sampled tree. Soil samples were stored as described previously as plant tissue samples. (3) Groundwater samples: The spring water (Miao Ling spring) near the sampling plots was used as the substitute for groundwater (Liu et al., 2017). The spring water samples were collected on the same day of the plant and soil sample collections. Samples were collected into 50‐ml vials, sealed with Parafilm “M” (Bemis NA Co.), placed in a portable freezer before transported to the laboratory and frozen at −20°C until isotopic examination.

Water extraction and isotopic analysis. The δ^18^O and δD values of water from different samples were examined at the Laboratory of Eco‐Hydrological Process and Mechanism, Beijing Forestry University. At the laboratory, one set of soil samples was measured for gravimetric water content from the sample weight loss by drying samples at 105°C for 48 hr. Water was extracted from the remaining soil samples and plant tissue samples using a cryogenic vacuum distillation method (West, Patrickson, & Ehleringer, 2006).

The isotopic compositions of all water samples were measured with a Liquid Water Isotope Analyzer (LWIA, DLT‐100; Los Gatos Research Inc., San Jose, USA) at the laboratory. Both δ^18^O and δD values can be measured simultaneously with this instrument. Isotope ratios are expressed as:

(6) $$\delta^{18}O\;\text{or}\;\delta D\;\left(‰\right)=\left(R_{\mathrm{sample}}/R_{\mathrm{standard}}-1\right)\times 1,000$$

where *R*_sample_ and *R*_*s*tandard_ indicate the isotope ratio of the samples and standard (Vienna Standard Mean Ocean Water). The measurement accuracies of δD and δ^18^O were ±0.3‰ and ±0.1‰, respectively.

### 2.5 Statistical analysis

Regressions between transpiration and environmental factors were plotted and estimates of *r* ^2^ and slope *p*‐values were performed in Sigmaplot 12.5 (SPSS Inc., Chicago, IL, USA).

The comparison of δ^18^O composition between plant tissue samples and soil water yielded information as to which layer was the water source.

To discover the possible sources of water uptake by plants, isotopic compositions of plant water samples was first compared with the water sources of soil of different depths (0–10, 10–20, 20–30, 30–40 and 40‐60 cm) and groundwater. According to the principle of isotope mass balance, the isotopic compositions of all potential water sources and plant water were input to IsoSource mixing model (Phillips & Gregg, 2003) approach to assess the contributions of different water sources used by plants. The fractional increment was set at 1%, and the uncertainty level was set at 0.2‰. Significant differences were determined via independent‐samples *t* test. This approach analyzed the data more systematically and provided a more quantitative assessment of the estimated range of feasible contributions of potential water sources to total water uptake (Jia, Yu, & Deng, 2013). It can calculate as many as ten water sources. The IsoSource mixing model was expressed with formula as follows:

(7) $$\begin{array}{cc} \delta Y\;=\; & c_{1}\delta X_{1}+c_{2}\delta X_{2}+c_{3}\delta X_{3}+\ldots +c_{n}\delta X_{n}\\ & c_{1}+c_{2}+c_{3}+\ldots +c_{n}=1 \end{array}$$

where *Y* is the ^18^O or D of water in plant, *X*_1_, *X*_2_, *X*_3_…*X*_*n*_ are the ^18^O or D of different water sources, and *c*_1_, *c*_2_, *c*_3_, *c*_*n*_ are the contribution rates of different water sources. *n* is number of water sources and in this study, *n* is six (soil water: 0–10, 10–20, 20–30, 30–40, and 40–60 cm and groundwater).

## 3 RESULTS

### 3.1 Environmental factors

**Figure 1:**
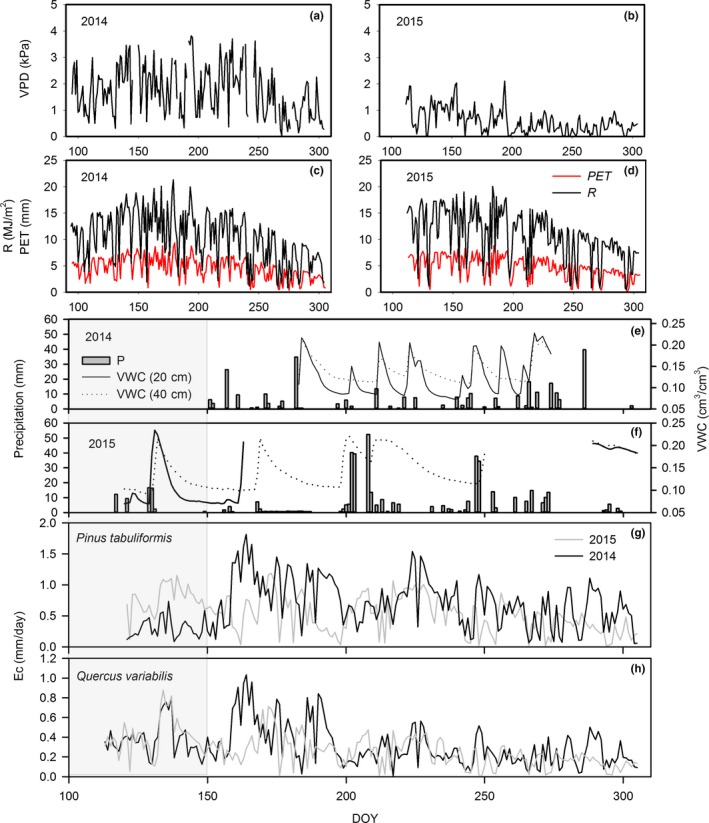
Mean daytime vapor pressure deficit (VPD), solar radiation (*R*), and potential evapotranspiration (PET) (a) ~ (d), as well as precipitation and soil water content (VWC) (e) and (f) along with daily transpiration of the two species (g) and (h) in 2014 and 2015. The shaded area indicates the beginning of the growing season with contrasting rainfall in the 2 years

The average daily VPD was higher in 2014 (1.67 kPa) than in 2015 (0.58 kPa), with the highest values recorded in August 2014 (3.81 kPa) and June 2015 (2.11 kPa) (Figure 1a,b). The average solar radiation (*R*) value was similar (*p* = .52) in the 2 years, that is, 10.48 and 10.90 MJ/m^2^ in 2014 and 2015, respectively. Potential evapotranspiration in both years was also similar, 4.59 and 4.78 mm in 2014 and 2015, respectively. In contrast to 2015, there was no rainfall from the beginning of the observation until DOY 150 in 2014. Approximately 70% of the total precipitation events occurred in July and August (Figure 1c,d shaded area). Weak precipitation events (5 mm or less) were frequent, accounting for 77.5% and 80.82% of total events in 2014 and 2015, respectively, while strong precipitation events (more than 10 mm) were infrequent, accounting for 22.5% and 19.18% of total events in 2014 and 2015, respectively. The temporal variations in soil water content are shown in Figure 1c,d. Rapid depletion of the soil water content could be observed during the rainless interval, when the high air temperature induced a high water demand by the trees. The soil water content decreased gradually after the precipitation events and remained at a low value until the next rainfall event that initiated a renewed cycle of soil water.

### 3.2 Canopy transpiration (*E*_c_), sap flow (*J*_s_), and canopy conductance (*G*_c_)

Canopy transpiration of the two species demonstrated noticeable differences in the 2 years of contrasting rainfall patterns (Figure 1e,f). The differences in transpiration of Pt in the 2 years were more obvious than those of Qv, especially at the beginning of the growing season (Figure 1 shadow area). There was no rainfall at the beginning of the growing season in 2014. Correspondingly, *E*_c_ of Pt was significantly lower than that in 2015 when rainfall reached 58.6 mm at the beginning of the growing season. By contrast, *E*_c_ of Qv during this period was similar in both years. *E*_c_ of Pt in 2015 was higher than that in 2014. By contrast, *E*_c_ of Qv in the 2 years was similar in magnitude.

**Figure 2:**
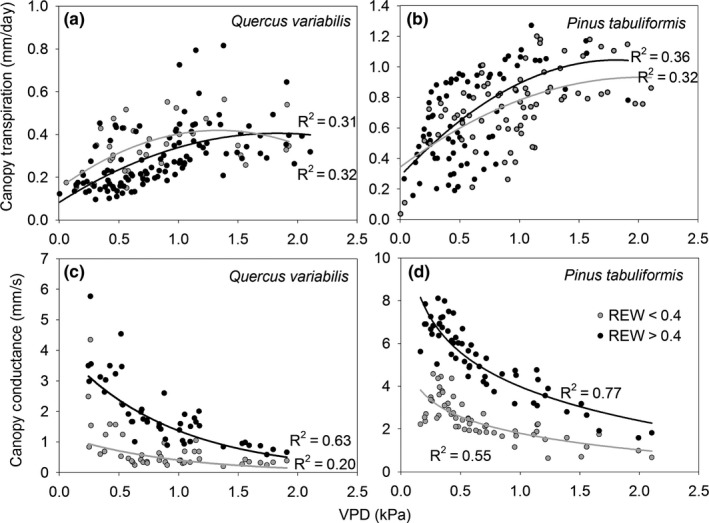
Relationship between canopy transpiration and vapor pressure deficit (VPD) (a) and (b) and between canopy conductance and VPD (c) and (d). Relative extractable soil water below 0.4 has been widely considered as a soil water stress condition (Bréda et al., 2006; Granier et al., 1999)

Canopy transpiration (*E*_c_) increased with VPD and gradually reached a maximum level (Figure 2a,b) except for Qv under the condition of REW < 0.4. The canopy conductance decreased logarithmically with VPD (Figure 2c,d). This suggests that the species regulated their conductance in response to high VPD. At a given VPD, *G*_c_ response to VPD was more sensitive when REW > 0.4 than when REW < 0.4.

**Figure 3:**
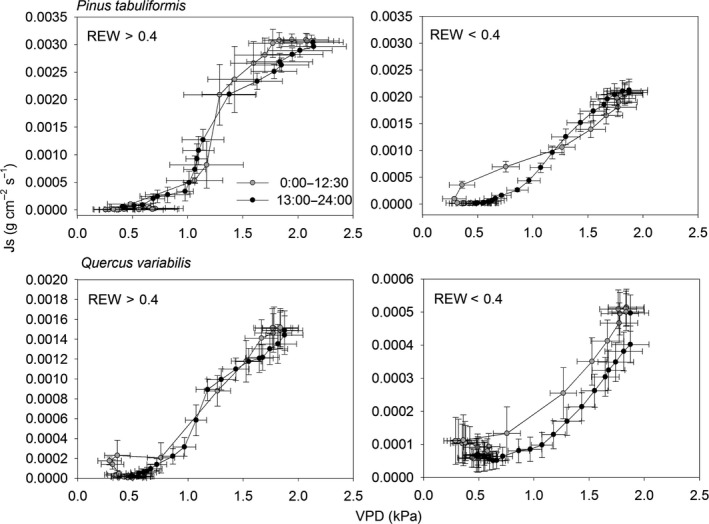
Hysteresis loop of the relationship between transpiration and vapor pressure deficit on a daily scale

**Figure 4:**
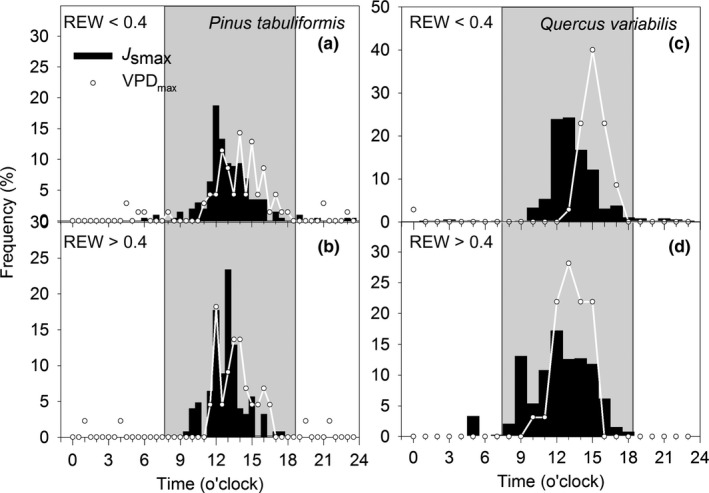
Frequency distribution of *J*_s_ (black bars) and vapor pressure deficit (dotted lines) peaks in different hours of a day

The influence of soil water on *E*_c_ can be observed when half‐hourly values of *J*_s_ and VPD are shown for bright summer days of similar average daily *R* and VPD. Under all conditions, plots of *J*_s_ against VPD yielded hysteresis loops (Figure 3) where *J*_s_ generally followed daily trends in VPD: *J*_s_ increased after sunrise, reached a maximum at noon, and declined until evening. The loop was considerably more pronounced for Qv when REW < 0.4 as compared to when REW > 0.4. Moreover, *J*_s_ of Qv was more than three times lower when REW<0.4 as compared to under the conditions of REW > 0.4 (Figure 3). Peak *J*_s_ of Qv tended to occur more concentrated before maximum VPD when REW < 0.4, while the peak *J*_s_ of Pt tended to occur distributed more evenly with highest diurnal VPD (Figure 4).

### 3.3 Contributions of the water sources used for plant uptake during water stress

**Figure 5:**
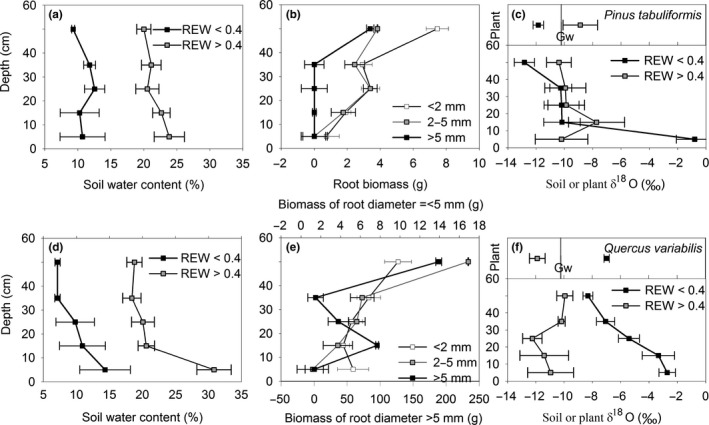
Oxygen isotopic composition (δ^18^O) of soil water (a) and (d) in comparison with that of plant and groundwater (Gw, showed as the bars in the lower panel of c and f) accompanied by the soil water content (b) and (e) and root biomass (c) and (f) throughout the soil profile during the rainy and dry season. Refer to (a), (b), and (c) for Pt and (d), (e), and (f) for Qv

The soil water of both species measured together with isotopic sampling was lower when REW < 0.4 than when REW > 0.4 (Figure 5a,d). Specifically, the average water content of Pt was 23.84% and 10.78% when REW > 0.4 and REW < 0.4, respectively, and decreased sinuously downward with the exception of increases at 12% at a depth of 25 cm when REW < 0.4, and at 22.1% and 22.85% at depths of 35 and 70 cm when REW > 0.4. A similar downward decrease was observed for Qv but without occasional increases. The average soil water content ranged from 7.14% to 14.33% when REW < 0.4 and from 18.78% to 30.83% when REW > 0.4. Large standard errors of SWC near the soil surface were observed, which reflects the considerable heterogeneity of the soil water content and indicates complex effects of evaporative demand and rainfall. The water content of the lowest soil layer remained relatively constant down to the maximum sampling depth. The root biomass showed that the roots of all classes were most abundant at 40–60 cm for both species (Figure 5b,e).

The observed ranges on earth of δ^18^O are much smaller than δD (Dawson & Siegwolf, 2007), which may lead to more accurate or at least equal result using δ^18^O. And due to a local meteoric water line (Jia et al., 2013), which reflects the relationship between δ^18^O and δD, these two isotopic compositions are closely related (*R* = 0.90, *n* = 35). So, it could show the same patterns or results by either δ^18^O or δD. In fact, either δ^18^O or δD can have the same results of plant water utilization (Snyder & Williams, 2000). So, δ^18^O was adopted in this study. The δ^18^O of soil water under both species showed contrasting patterns under different water stress conditions (Figure 5c,f). For Pt, comparatively high values of −0.8‰ at the soil surface were observed when REW < 0.4, followed by a decrease to −10‰ between the depths of 20–30 and 40–60 cm, and a further deduction to the value of −13‰. When REW > 0.4, only the surface and bottom layers were split from the curve of REW < 0.4 but both stayed at around −10‰. For Qv, the isotopic composition of soil water in different layers was distinct at different periods. δ^18^O decreased continuously from −2.5‰ at the surface to −8.3‰ at the bottom when REW* *< 0.4. By contrast, the isotopic values of different layers varied within the range of −12‰ to −10‰ when REW > 0.4.

Xylem δ^18^O of both species crossed with that of the soil water at depths between 20 and 40 cm when REW > 0.4 and below 40 cm when REW* *< 0.4 (Figure 5c,f). The groundwater remained at a stable value of −10.21‰ ± 0.17‰ (*SD*) (presented as Gw in Figure 5c,f), which was significantly different from the isotopic compositions of both species (*p* = .589, Paired *t* test).

**Figure 6:**
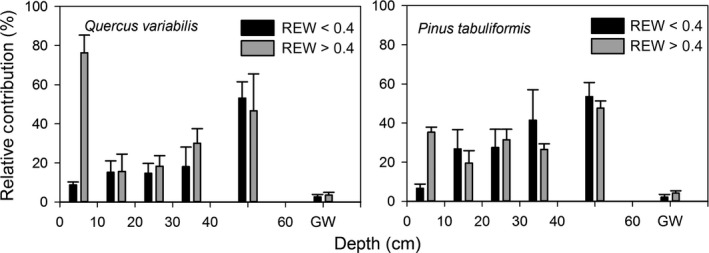
Proportions of water taken up from different layers based on the examined soil profile for Qv and Pt under different soil water conditions. GW stands for groundwater

The IsoSource mixing model revealed a variety of mixtures from the possible water sources that contributed to total plant water uptake (Figure 6). The percent contribution from each depth ranged from 2.2% to 76.2%. Compared to REW < 0.4, the relative contribution of shallow soil water increased greatly when REW > 0.4 for both species. However, deep soil water (40–60 cm) contributed to transpiration under all soil water conditions (Figure 6).

## 4 DISCUSSION

### 4.1 Indirect influence of rainfall pattern on transpiration

Theoretically, transpiration should increase with rainfall due to an increased soil water supply. For example, a higher stand transpiration rate after as opposed to before precipitation was observed under similar VPD and *R* (Shen, Gao, Fu, & Lü, 2015). In our study, this was true for Pt at the beginning of the growing season in 2015. However, high rainfall did not induce an increase in total *E*_c_ of both species in the growing season in 2015. Contrary to distinct rainless intervals in other studies, the continuous rainfall events in combination with total rainfall increased the relative humidity, which consequently decreased the VPD (an average daytime value of 0.68 ± 0.5 kPa in 2015 in comparison with 1.0 ± 0.7 kPa in 2014). We proved that stand transpiration was significantly influenced by VPD under all soil water conditions (Figure 2). Thus, low VPD would pose limitation over the transpiration. Previous studies proved the influence of precipitation patterns on tree response to transient soil moisture following precipitation pulses (O'Grady et al., 2008; Plaut et al., 2013). In this study, we also proved that rainfall patterns exerted influence over *E*_c_ by causing changes to VPD.

### 4.2 Different water use strategies by the two species

Both species demonstrated saturation of *E*_c_ approaching high VPD (Figure 2a,b). This can be primarily attributed to a decrease in *G*_c_ with increasing VPD (Figure 2c,d), which has also been observed by others (Wieser & Leo, 2012; Zimmermann et al., 2000). However, the two species demonstrated different transpiration and canopy conductance patterns in response to VPD under contrasting soil water conditions, and this would be attributed to their xylem anatomy and water use strategies. Several studies have found that oaks and other ring‐porous species tend to be more anisohydric, exhibiting increasingly more negative leaf water potentials with increasing evaporative demand and drought stress (Renninger et al., 2015). However, both species were found to have similar behavior (Martínez‐Vilalta et al., 2014) and considered “partial isohydric” in a synthesis study to compare directly isohydric/anisohydric behavior across species and ecosystem types (Martínez‐Vilalta et al., 2014). Therefore, although differences in anatomy and leaf habit are evident between pines and oaks, results from previous studies are mixed with regard to how these differences will affect physiological functioning of each genus in a resource‐limited environment.

The isohydric/anisohydric water use behavior can not only be inferred from leaf level data but also the tree‐level regulation of water use (Kumagai & Porporato, 2012). In our study, Qv appeared to adopt a relatively anisohydric water use strategy as its transpiration and canopy conductance remained the same range at REW < 0.4 as that at REW > 0.4 (Figures 1h and 2). In comparison to Pt, the larger conduit of Qv and anisohydric water use strategy render this species more vulnerable to cavitation. First, *E*_c_ of Qv decreased instead of sustained the high level in response to high VPD when REW < 0.4 (Figure 2a). Second, the probability of diurnal peak *E*_c_ was concentrated when REW < 0.4 (Figure 4). It indicated that the water status and transport capacity of Qv were jeopardized by cavitation and unable to sustain the high‐level *E*_c_ under high VPD. Third, aggravated hysteresis also indicated the possibility of cavitation. Hysteresis in the relationship between *E*_c_ and VPD has been observed in other studies (Meinzer et al., 1999; Zeppel, Murray, Barton, & Eamus, 2004). Increased hysteresis under water stress can be attributed to increasing stomatal control of transpiration which is explained by the loss of hydraulic conductance, such as cavitation (O'Grady et al., 2008). By contrast, Pt demonstrated relatively high *E*_c_ and *G*_c_ throughout the whole growing season, but it decreased transpiration under less ideal water conditions, such as at the beginning of the growing season with contrasting rainfall amount (in 2014 vs. 2015) (Figure 1g) and when REW < 0.4 (Figure 2b,d). These physiological drought adaptation mechanisms fit a typical water‐saving strategy (Brito et al., 2015). Exposed to water stress, the first response of trees is stomatal control. Decreased canopy conductance caused by stomatal closure due to increasing VPD and soil drought has been found in many species (Gieger & Leuschner, 2004; Peters, Gonzalez‐Rodriguez, Jiménez, Morales, & Wieser, 2008). This would prevent transpiration declining to values that could reduce plant water potential such that the xylem water column undergoes catastrophic cavitation (Meinzer & McCulloh, 2013). Moreover, the xylem tracheids of small diameter of Pt are resistant to cavitation. In terms of stomatal regulation of water transport, conifers are found to have the largest “safety margin” between minimum in situ water potential and the water potential at which 50% of hydraulic conductivity is lost to embolism in a global synthesis study (Choat et al., 2012). Therefore, the hysteresis loop of this species did not enlarge when REW < 0.4 (Figure 3).

Dated back to 1950s, the forests in this mountain area are of the same age. From the perspective of transpiration, the individual sizes of the same species are similar, but vary between species. Because tree diameter is directly linked to the size of water‐conducting area of trees, such differences are partial reason to the differences of total stand transpiration between the two species (Figures 1 and 2). From the perspective of water uptake depths, studies have been inconsistent with relationship between tree diameter and water uptake depths. Bigger trees tended to take up soil water closer to the soil surface (Hardanto, Röll, & Hendrayanto‐Hölscher, 2017). By contrast, significant positive linear relationships of tree DBH and water uptake depths of canopy trees were reported in old‐growth tropical tree plantation (Andrade, Goldstein, Holbrook, Cavelier, & Wright, 1999). In another case, no correlations between water uptake depth and DBH were found in a young tropical tree plantation (Schwendenmann, Pendall, Sanchez‐Bragado, Kunert, & Hölscher, 2015). Such inconsistency may be attributed to the fact that there is no fixed relationship between tree diameter and root distribution (Snyder, 2007), but soil water uptake among species from different soil layers was proved primarily to be the result of differential root depth and architecture (Trogisch, Salmon, He, Hector, & Scherer‐Lorenzen, 2016). In our study, the distribution of root system would also explain the water uptake depth and the transpiration differences. The root biomass of Qv was much higher than that of Pt, especially in the surface layers (0–25 cm), which were rapidly replenished after rainfall. Shallow distributions of root systems may be a reason for sensitive responses to soil moisture recovery (Kume et al., 2007). Therefore, quick replenishment by Qv was able to alleviate cavitation after rainless intervals and to maintain relatively stable transpiration for the whole growing season. By contrast, Pt demonstrated only one peak of root biomass at the depth of 40–60 cm, and thus relied mainly on water supply from deeper layers that were less subject to evaporation.

### 4.3 Acquisition of water sources and implications

The xylem water δ^18^O value of both species overlapped with the δ^18^O value of the deeper layers when soil water condition was less stressed when REW > 0.4 (Figure 5c,f). Moreover, the results from the IsoSource mixing model also revealed that both species utilized a substantial amount of water stored within the deeper, wetter layers (40–60 cm) during the whole growing season under conditions of either REW > 0.4 or REW < 0.4 (Figure 6). The ability to access deep soil water may reduce vulnerability to drought, and this seems particularly important for the survival of evergreen trees that must cope with long‐lasting soil drought, similar to observations in semi‐arid environments (Lubczynski, 2009). Due to IsoSource mixing model calculations, groundwater may contribute to plant water uptake (Figure 6), the results could be misinterpreted though. First, the isotopic compositions of both species were significantly different from the groundwater (*p* = .589, Paired *t* test). Second, the distribution of plant roots is an important factor in determining the depth of plant water use. The relative distribution and activity of surface and deep roots affect the range of water absorbed by plants (Yu, Jie, Nakayama, & Yan, 2007). The depth of groundwater in this area is below 23 m, which is much deeper than the distribution depth (~60 cm) of both species in this study. This indicated that the trees did not use groundwater for plant utilization and transpiration, which was consistent with our previous research in this area. So, even the IsoSource mixing model provides a feasible method for quantify plant water uptake from different possible water sources, the results should be interpreted according to the actual conditions (Rothfuss & Javaux, 2017) such as root distribution.

Even with the advantage of a deep root system, conclusive predictions cannot be made regarding the influence of water acquisition strategy on the survival of a species in the future. From the perspective of water accessibility, the survival of a species will be a product of the type of drought shaped by rainfall pattern, combined with the configuration of root systems in different soil layers. Precipitation projections feature great uncertainty (Taylor, de Jeu, Guichard, Harris, & Dorigo, 2012), and rainfall events of different magnitudes infiltrate different soil layers. Thus, the predicated future alteration of precipitation regimes over long periods would change the current soil water profile for coexisting species. However, it is insufficient to determine the future composition and structure of the community based only on the projected rainfall pattern because the configuration of the root system in different soil layers determines the capacity of trees to use rainfall pulses.

## 5 CONCLUSION

Throughout the observation periods, Pt demonstrated higher transpiration/canopy conductance than Qv did. This can be seen as the result of different biophysiological response of the two species to water stress. Pt was more responsive to soil water stress by adopting strict stomatal control over transpiration. By contrast, Qv was more prone to adopt anisohydric water use strategy when soil water stress increased. However, the water use strategy of both species comprised access to deep‐layer water at this site, but Qv was more efficient to use surface water than Pt due to developed surface root system. The results have implications for forest management in ecologically degraded and drought‐prone areas, the selection of sustainable reforestation species, and the development of regional hydrological models.

## CONFLICT OF INTEREST

None declared.

## AUTHORS CONTRIBUTIONS

Guodong Jia contributed to the literature search, study design, data interpretation, and manuscript writing and revision. Ziqiang Liu contributed to the fieldwork and data analysis. Lixin Chen contributed to data processing and manuscript drafting. Xinxiao Yu contributed to study design and manuscript revision.

## References

[ece33584-bib-0001] Allen, C. D. , Macalady, A. K. , Chenchouni, H. , Bachelet, D. , McDowell, N. , Vennetier, M. , … Hogg, E. T. (2010). A global overview of drought and heat‐induced tree mortality reveals emerging climate change risks for forests. Forest Ecology and Management, 259, 660–684. https://doi.org/10.1016/j.foreco.2009.09.001

[ece33584-bib-0002] Andrade, J. L. , Goldstein, G. , Holbrook, N. M. , Cavelier, J. , & Wright, S. J. (1999). Partitioning of soil water among canopy trees in a seasonally dry tropical forest. Oecologia, 121, 293–301. https://doi.org/10.1007/s004420050931 28308316 10.1007/s004420050931

[ece33584-bib-0003] Bréda, N. , Huc, R. , Granier, A. , & Dreyer, E. (2006). Temperate forest trees and stands under severe drought: A review of ecophysiological responses, adaptation processes and long‐term consequences. Annals of Forest Science, 63, 625–644. https://doi.org/10.1051/forest:2006042

[ece33584-bib-0004] Brito, P. , Lorenzo, J. R. , González‐Rodríguez, Á. M. , Morales, D. , Wieser, G. , & Jiménez, M. S. (2015). Canopy transpiration of a semi arid *Pinus canariensis* forest at a treeline ecotone in two hydrologically contrasting years. Agricultural and Forest Meteorology, 201, 120–127. https://doi.org/10.1016/j.agrformet.2014.11.008

[ece33584-bib-0005] Choat, B. , Jansen, S. , Brodribb, T. J. , Cochard, H. , Delzon, S. , Bhaskar, R. , … Hacke, U. G. (2012). Global convergence in the vulnerability of forests to drought. Nature, 491, 752–755. https://doi.org/10.1038/nature11688 23172141 10.1038/nature11688

[ece33584-bib-0006] Dawson, T. E. , Mambelli, S. , Plamboeck, A. H. , Templer, P. H. , & Tu, K. P. (2002). Stable isotopes in plant ecology. Annual Review of Ecology and Systematics, 33, 507–559. https://doi.org/10.1146/annurev.ecolsys.33.020602.095451

[ece33584-bib-0007] Dawson, T. E. , & Siegwolf, R. T. (2007). Using stable isotopes as indicators, tracers, and recorders of ecological change: Some context and background. Terrestrial Ecology, 1, 1–18. https://doi.org/10.1016/s1936-7961(07)01001-9

[ece33584-bib-0008] Domec, J. C. , King, J. S. , Ward, E. , Oishi, A. C. , Palmroth, S. , Radecki, A. , … Johnson, D. M. (2015). Conversion of natural forests to managed forest plantations decreases tree resistance to prolonged droughts. Forest Ecology and Management, 355, 58–71. https://doi.org/10.1016/j.foreco.2015.04.012

[ece33584-bib-0009] Ford, C. R. , McGuire, M. A. , Mitchell, R. J. , & Teskey, R. O. (2004). Assessing variation in the radial profile of sap flux density in *Pinus* species and its effect on daily water use. Tree Physiology, 24, 241–249. https://doi.org/10.1093/treephys/24.3.241 14704134 10.1093/treephys/24.3.241

[ece33584-bib-0010] Forrester, D. I. , Theiveyanathan, S. , Collopy, J. J. , & Marcar, N. E. (2010). Enhanced water use efficiency in a mixed *Eucalyptus globulus* and *Acacia mearnsii* plantation. Forest Ecology and Management, 259, 1761–1770. https://doi.org/10.1016/j.foreco.2009.07.036

[ece33584-bib-0011] Gartner, K. , Nadezhdina, N. , Englisch, M. , Cermak, J. , & Leitgeb, E. (2009). Sap flow of birch and Norway spruce during the European heat and drought in summer 2003. Forest Ecology and Management, 258, 590–599. https://doi.org/10.1016/j.foreco.2009.04.028

[ece33584-bib-0012] Gieger, T. , & Leuschner, C. (2004). Altitudinal change in needle water relations of *Pinus canariensis* and possible evidence of a drought‐induced alpine timberline on Mt. Teide, Tenerife. Flora‐Morphology, Distribution, Functional Ecology of Plants, 199, 100–109. https://doi.org/10.1078/0367-2530-00139

[ece33584-bib-0013] Granier, A. (1987). Evaluation of transpiration in a Douglas‐fir stand by means of sap flow measurements. Tree Physiology, 3, 309–320. https://doi.org/10.1093/treephys/3.4.309 14975915 10.1093/treephys/3.4.309

[ece33584-bib-0014] Granier, A. , Bréda, N. , Biron, P. , & Villette, S. (1999). A lumped water balance model to evaluate duration and intensity of drought constraints in forest stands. Ecological Modelling, 116, 269–283. https://doi.org/10.1016/S0304-3800(98)00205-1

[ece33584-bib-0015] Hardanto, A. , Röll, A. , & Hendrayanto‐Hölscher, D. (2017). Tree soil water uptake and transpiration in mono‐cultural and jungle rubber stands of Sumatra. Forest Ecology & Management, 397, 67–77. https://doi.org/10.1016/j.foreco.2017.04.032

[ece33584-bib-0016] Hultine, K. , Nagler, P. , Morino, K. , Bush, S. , Burtch, K. , Dennison, P. E. , … Ehleringer, J. R. (2010). Sap flux‐scaled transpiration by tamarisk (*Tamarix* spp.) before, during and after episodic defoliation by the saltcedar leaf beetle (*Diorhabda carinulata*). Agricultural and Forest Meteorology, 150, 1467–1475. https://doi.org/10.1016/j.agrformet.2010.07.009

[ece33584-bib-0017] Jactel, H. , Nicoll, B. C. , Branco, M. , Gonzalez‐Olabarria, J. R. , Grodzki, W. , Långström, B. , … Piou, D. (2009). The influences of forest stand management on biotic and abiotic risks of damage. Annals of Forest Science, 66, 1–18.

[ece33584-bib-0018] Jia, G. , Yu, X. , & Deng, W. (2013). Seasonal water use patterns of semi‐arid plants in China. The Forestry Chronicle, 89, 169–177. https://doi.org/10.5558/tfc2013-034

[ece33584-bib-0019] Kumagai, T. O. , & Porporato, A. (2012). Strategies of a Bornean tropical rainforest water use as a function of rainfall regime: Isohydric or anisohydric? Plant, Cell & Environment, 35, 61–71. https://doi.org/10.1111/j.1365-3040.2011.02428.x 10.1111/j.1365-3040.2011.02428.x 21933196

[ece33584-bib-0020] Kume, T. , Takizawa, H. , Yoshifuji, N. , Tanaka, K. , Tantasirin, C. , Tanaka, N. , & Suzuki, M. (2007). Impact of soil drought on sap flow and water status of evergreen trees in a tropical monsoon forest in northern Thailand. Forest Ecology and Management, 238, 220–230. https://doi.org/10.1016/j.foreco.2006.10.019

[ece33584-bib-0021] Liu, Z. , Yu, X. , Jia, G. , Jia, J. , Lou, Y. , & Lu, W. (2017). Contrasting water sources of evergreen and deciduous tree species in rocky mountain area of Beijing, China. Catena, 150, 108–115. https://doi.org/10.1016/j.catena.2016.11.013

[ece33584-bib-0022] Lu, P. , Urban, L. , & Zhao, P. (2004). Granier's thermal dissipation probe (TDP) method for measuring sap flow in trees: Theory and practice. Acta Botanica Sinica, 46, 631–646.

[ece33584-bib-0023] Lubczynski, M. (2009). The hydrogeological role of trees in water‐limited environments. Hydrogeology Journal, 17, 247–259. https://doi.org/10.1007/s10040-008-0357-3

[ece33584-bib-0024] Martínez‐Vilalta, J. , Poyatos, R. , Aguadé, D. , Retana, J. , & Mencuccini, M. (2014). A new look at water transport regulation in plants. New Phytologist, 204, 105–115. https://doi.org/10.1111/nph.12912 24985503 10.1111/nph.12912

[ece33584-bib-0025] Matheny, A. M. , Bohrer, G. , Vogel, C. S. , Morin, T. H. , He, L. , Frasson, R. P. D. M. , … Ivanov, V. Y. (2014). Species‐specific transpiration responses to intermediate disturbance in a northern hardwood forest. Journal of Geophysical Research: Biogeosciences, 119, 2292–2311. https://doi.org/10.1002/2014jg002804

[ece33584-bib-0026] McCulloh, K. , Sperry, J. S. , Lachenbruch, B. , Meinzer, F. C. , Reich, P. B. , & Voelker, S. (2010). Moving water well: Comparing hydraulic efficiency in twigs and trunks of coniferous, ring‐porous, and diffuse‐porous saplings from temperate and tropical forests. New Phytologist, 186, 439–450. https://doi.org/10.1111/j.1469-8137.2010.03181.x 20158616 10.1111/j.1469-8137.2010.03181.x

[ece33584-bib-0027] Meinzer, F. , Goldstein, G. , Franco, A. , Bustamante, M. , Igler, E. , Jackson, P. , … Rundel, P. (1999). Atmospheric and hydraulic limitations on transpiration in Brazilian cerrado woody species. Functional Ecology, 13, 273–282. https://doi.org/10.1046/j.1365-2435.1999.00313.x

[ece33584-bib-0028] Meinzer, F. C. , & McCulloh, K. A. (2013). Xylem recovery from drought‐induced embolism: Where is the hydraulic point of no return? Tree Physiology, 33, 331–334. https://doi.org/10.1093/treephys/tpt022 23612243 10.1093/treephys/tpt022

[ece33584-bib-0029] Mielke, M. S. , Oliva, M. A. , Nfde, B. , Penched, R. M. , Martinez, C. A. , & Acde, A. (1999). Stomatal control of transpiration in the canopy of a clonal *Eucalyptus grandis* plantation. Trees, 13, 152–160. https://doi.org/10.1007/pl00009746

[ece33584-bib-0030] Nowacki, G. J. , & Abrams, M. D. (2008). The demise of fire and “mesophication” of forests in the eastern United States. BioScience, 58, 123–138. https://doi.org/10.1641/B580207

[ece33584-bib-0031] O'Grady, A. P. , Worledge, D. , & Battaglia, M. (2008). Constraints on transpiration of *Eucalyptus globulus* in southern Tasmania, Australia. Agricultural and Forest Meteorology, 148, 453–465. https://doi.org/10.1016/j.agrformet.2007.10.006

[ece33584-bib-0032] Peters, J. , Gonzalez‐Rodriguez, A. , Jiménez, M. , Morales, D. , & Wieser, G. (2008). Influence of canopy position, needle age and season on the foliar gas exchange of *Pinus canariensis* . European Journal of Forest Research, 127, 293–299. https://doi.org/10.1007/s10342-008-0205-y

[ece33584-bib-0033] Phillips, D. L. , & Gregg, J. W. (2003). Source partitioning using stable isotopes: Coping with too many sources. Oecologia, 136, 261–269. https://doi.org/10.1007/s00442-003-1218-3 12759813 10.1007/s00442-003-1218-3

[ece33584-bib-0034] Plaut, J. A. , Wadsworth, W. D. , Pangle, R. , Yepez, E. A. , McDowell, N. G. , & Pockman, W. T. (2013). Reduced transpiration response to precipitation pulses precedes mortality in a pinon–juniper woodland subject to prolonged drought. New Phytologist, 200, 375–387. https://doi.org/10.1111/nph.12392 23844951 10.1111/nph.12392

[ece33584-bib-0035] Renninger, H. J. , Carlo, N. J. , Clark, K. L. , & Schäfer, K. V. (2015). Resource use and efficiency, and stomatal responses to environmental drivers of oak and pine species in an Atlantic Coastal Plain forest. Frontiers in Plant Science, 6, 297 https://doi.org/10.3389/fpls.2015.00297 25999966 10.3389/fpls.2015.00297 PMC4423344

[ece33584-bib-0036] Rothfuss, Y. , & Javaux, M. (2017). Reviews and syntheses: Isotopic approaches to quantify root water uptake: A review and comparison of methods. Biogeosciences, 14, 2199–2224. https://doi.org/10.5194/bg-14-2199-2017

[ece33584-bib-0037] Schwendenmann, L. , Pendall, E. , Sanchez‐Bragado, R. , Kunert, N. , & Hölscher, D. (2015). Tree water uptake in a tropical plantation varying in tree diversity: Interspecific differences, seasonal shifts and complementarity. Ecohydrology, 8, 1–12. https://doi.org/10.1002/eco.v8.1

[ece33584-bib-0038] Shen, Q. , Gao, G. , Fu, B. , & Lü, Y. (2015). Responses of shelterbelt stand transpiration to drought and groundwater variations in an arid inland river basin of Northwest China. Journal of Hydrology, 531, 738–748. https://doi.org/10.1016/j.jhydrol.2015.10.053

[ece33584-bib-0039] Shi, P. , Sun, X. , Wang, M. , Li, N. , Wang, J. , Jin, Y. , … Yin, W. (2014). Climate change regionalization in China (1961–2010). Science China: Earth Sciences, 44, 2294–2306.

[ece33584-bib-0040] Snyder, K. (2007). Root allocation and water uptake patterns in riparian tree saplings: Responses to irrigation and defoliation in a glasshouse environment. Forest Ecology & Management, 246, 222–231. https://doi.org/10.1016/j.foreco.2007.04.032

[ece33584-bib-0041] Snyder, K. A. , & Williams, D. G. (2000). Water sources used by riparian trees varies among stream types on the San Pedro River, Arizona. Agricultural and Forest Meteorology, 105, 227–240. https://doi.org/10.1016/S0168-1923(00)00193-3

[ece33584-bib-0042] Taylor, C. M. , de Jeu, R. A. , Guichard, F. , Harris, P. P. , & Dorigo, W. A. (2012). Afternoon rain more likely over drier soils. Nature, 489, 423–426. https://doi.org/10.1038/nature11377 22972193 10.1038/nature11377

[ece33584-bib-0043] Tognetti, R. , Giovannelli, A. , Lavini, A. , Morelli, G. , Fragnito, F. , & d'Andria, R. (2009). Assessing environmental controls over conductances through the soil‐plant‐atmosphere continuum in an experimental olive tree plantation of southern Italy. Agricultural and Forest Meteorology, 149, 1229–1243. https://doi.org/10.1016/j.agrformet.2009.02.008

[ece33584-bib-0044] Trogisch, S. , Salmon, Y. , He, J. S. , Hector, A. , & Scherer‐Lorenzen, M. (2016). Spatio‐temporal water uptake patterns of tree saplings are not altered by interspecific interaction in the early stage of a subtropical forest. Forest Ecology & Management, 367, 52–61. https://doi.org/10.1016/j.foreco.2016.02.018

[ece33584-bib-0045] West, A. G. , Patrickson, S. J. , & Ehleringer, J. R. (2006). Water extraction times for plant and soil materials used in stable isotope analysis. Rapid Communications in Mass Spectrometry RCM, 20, 1317 https://doi.org/10.1002/(ISSN)1097-0231 16555369 10.1002/rcm.2456

[ece33584-bib-0046] Wieser, G. , & Leo, M. (2012). Whole‐tree water use by *Pinus cembra* at the treeline in the Central Tyrolean Alps. Plant Ecology & Diversity, 5, 81–88. https://doi.org/10.1080/17550874.2012.688070

[ece33584-bib-0047] Wilson, K. B. , Hanson, P. J. , Mulholland, P. J. , Baldocchi, D. D. , & Wullschleger, S. D. (2001). A comparison of methods for determining forest evapotranspiration and its components: Sap‐flow, soil water budget, eddy covariance and catchment water balance. Agricultural and Forest Meteorology, 106, 153–168. https://doi.org/10.1016/S0168-1923(00)00199-4

[ece33584-bib-0048] Yu, G. R. , Jie, Z. , Nakayama, K. , & Yan, J. (2007). Root water uptake and profile soil water as affected by vertical root distribution. Plant Ecology, 189, 15–30. https://doi.org/10.1007/s11258-006-9163-y

[ece33584-bib-0049] Zeppel, M. , Macinnis‐Ng, C. , Palmer, A. , Taylor, D. , Whitley, R. , Fuentes, S. , … Eamus, D. (2008). An analysis of the sensitivity of sap flux to soil and plant variables assessed for an Australian woodland using a soil–plant–atmosphere model. Functional Plant Biology, 35, 509–520. https://doi.org/10.1071/FP08114 10.1071/FP08114 32688807

[ece33584-bib-0050] Zeppel, M. J. , Murray, B. R. , Barton, C. , & Eamus, D. (2004). Seasonal responses of xylem sap velocity to VPD and solar radiation during drought in a stand of native trees in temperate Australia. Functional Plant Biology, 31, 461–470. https://doi.org/10.1071/FP03220 10.1071/FP03220 32688918

[ece33584-bib-0051] Zimmermann, R. , Schulze, E. D. , Wirth, C. , Schulze, E. E. , Mcdonald, K. C. , Vygodskaya, N. N. , & Ziegler, W. (2000). Canopy transpiration in a chronosequence of Central Siberian pine forests. Global Change Biology, 6, 25–37. https://doi.org/10.1046/j.1365-2486.2000.00289.x

